# Neural and Behavioral Correlates of Evidence Accumulation in Human Click-Based Echolocation

**DOI:** 10.1523/ENEURO.0342-25.2026

**Published:** 2026-04-14

**Authors:** Haydée G. García-Lázaro, Santani Teng

**Affiliations:** ^1^Smith-Kettlewell Eye Research Institute, San Francisco, California 94115; ^2^Cardiff University Brain Research Imaging Centre, School of Psychology, Cardiff University, Cardiff CF24 4HQ, United Kingdom

**Keywords:** blindness, echolocation, EEG, evidence accumulation, MVPA, psychophysics, spatial localization

## Abstract

Echolocation enables blind individuals to perceive and navigate their environment by emitting clicks and interpreting their returning echoes. While expert blind echolocators demonstrate remarkable spatial accuracy, the behavioral and neural mechanisms by which spatial echoacoustic cues are combined across repeated samples remain less explored. Here, we investigated the temporal dynamics of spatial information processing in human click-based echolocation using electroencephalography (EEG). Blind expert echolocators (*n* = 4, all males) and novice sighted participants (*n* = 21, 12 males) localized virtual spatialized echoes derived from realistic synthesized mouth clicks, presented in trains of 2–11 clicks. Behavioral results showed that blind expert echolocators significantly outperformed sighted controls in spatial localization. For these experts, localization thresholds decreased as the number of clicks increased, a pattern consistent with cumulative integration of spatial information across repeated samples. EEG decoding analyses revealed reliable neural discrimination of echo laterality from the first click that correlated with overall spatial localization performance. Across successive clicks, neural responses evolved systematically, reflecting sequence-position–dependent changes in neural dynamics. EEG trial-level modeling further allowed us to distinguish accumulation-consistent decision readout policies from alternative repetition-based accounts, revealing individual differences in decision policies among expert echolocators. These findings provide, to our knowledge, the first fine-grained account of the temporal neural dynamics supporting human click-based echolocation, directly linked to behavioral performance across multiple samples. They reveal how, in expert echolocators who successfully performed the task, successive echoes are progressively integrated into coherent spatial representations, demonstrating adaptive sensory processing in the absence of vision.

## Significance Statement

Remarkably, some blind individuals navigate the world using echolocation, producing mouth clicks and interpreting returning echoes to perceive their surroundings. Yet how the brain combines successive echoes to build spatial representations remains poorly understood. Here, we show that expert blind echolocators localized echoes more accurately than sighted novices and that their performance improved as additional clicks provided more spatial information over time. Brain recordings revealed that neural activity distinguished sound location from the earliest echoes and evolved systematically across click sequences in parallel with behavioral improvements. These findings provide a detailed account of how the human brain transforms repeated acoustic information into stable spatial representations, supporting navigation in the absence of vision.

## Introduction

Human echolocation is a remarkable form of active sensing that enables some blind individuals to perceive and navigate their environment using sound. By producing brief sounds, typically mouth clicks, and interpreting the echoes reflected from surrounding surfaces, echolocators extract spatial information to map their surroundings. Proficient blind echolocators consistently outperform nonexpert blind and sighted individuals in various echoacoustic tasks. These include the perception of object distance, size, shape, texture, location, density, and motion (Kellogg, 1962; Rice, 1969; Hausfeld et al., 1982; Schenkman and Nilsson, 2010, 2011; Teng et al., 2011, 2024; Teng and Whitney, 2011; Thaler et al., 2013; Kolarik et al., 2014; Milne et al., 2014). The spatial resolution achieved by blind echolocators often surpasses that of sighted individuals (Teng and Whitney, 2011), even when their learning rates are comparable (Teng and Whitney, 2011; Tonelli et al., 2016; Heller et al., 2017; Norman et al., 2021). This distinction suggests that the learning rate and spatial resolution likely reflect separate components of echolocation proficiency, shaped by different underlying mechanisms modulated by visual experience.

Beyond perceptual accuracy, proficient human echolocators dynamically modulate their sampling strategies to optimize spatial perception based on task demands and environmental complexity. For instance, they emit more and louder clicks under acoustically challenging conditions or when detecting and localizing objects at wider azimuths from the body midline (Norman and Thaler, 2018; Thaler et al., 2018, 2019), likely to enhance echo salience and signal-to-noise ratio (SNR). The target size also influences sampling behavior. When localizing smaller targets, echolocators engage in head-scanning movements that cover wider spatial areas, requiring more clicks and time before anchoring to near-target locations; in contrast, larger objects are typically localized with fewer clicks, reduced exploratory motion, and higher precision (Teng and Fusco, 2019; Patel et al., 2024). These adaptive behaviors suggest a flexible, goal-directed sampling process in which echolocators appear to gather more echoacoustic information over multiple clicks to refine their internal representations.

This dynamic modulation raises a fundamental computational question: does spatial perception in echolocation rely on a single optimal echo, or does it emerge from the cumulative integration of echoacoustic information gathered across multiple echoes? In audiovisual perception, sensory evidence accumulation frameworks describe how decisions emerge through the progressive integration of noisy inputs over time (Noppeney et al., 2010; Hanks et al., 2015; Yang et al., 2016; Yao et al., 2020; Pinto et al., 2022; Bondy et al., 2025). Whether a similar process governs echoacoustic spatial perception remains unknown. One possibility is that repeated clicks increase the chance of producing an optimal sample, which could occur at any point in a click sequence, without further benefit from repetitions and information integration. Alternatively, if cumulative echoacoustic integration is a fundamental process for echoacoustic perception, each additional sample should contribute incrementally to the refinement of a spatial percept. Distinguishing between these accounts is essential to understanding the principles by which humans extract spatial information through echolocation.

To address this question, here we investigated the neural and behavioral dynamics of echoacoustic spatial perception. Participants heard synthetic clicks and their spatialized reflections, simulating a virtual object located at varying eccentricities in the azimuth, and indicated whether the object was located to the left or right of the midline. Using electroencephalographic (EEG) recordings, we examined neural and behavioral responses while blind expert echolocators and sighted novice controls performed a spatial localization task requiring them to localize virtual objects based on spatialized echoes. We predicted that (1) localization performance would improve with increasing azimuthal eccentricity of the echo; (2) performance would improve with increasing numbers of echoacoustic samples, consistent with evidence accumulation rather than optimal single-sample processing; and (3) expert blind echolocators would demonstrate superior localization accuracy compared with novice sighted controls, with both behavioral and neural measures reflecting expertise-driven differences in spatial processing. Consistent with these predictions, blind experts improved localization accuracy with eccentricity and click count and outperformed novice sighted controls. EEG decoding revealed temporally structured neural representations of echo laterality that correlate with performance across the sequence and were detectable early in the trial. Trial-level computational modeling further indicated that click-count–dependent improvements were better explained by graded integration of neural evidence across clicks. Together, these findings support the idea that spatial representations in human echolocation evolve through cumulative integration of sensory information over time, shaped by echo perceptibility and by individual differences in neural readout policies.

## Materials and Methods

### Participants

In this study, 21 sighted individuals (mean age = 32.3 years; SD = 5.7 years, 12 males) and four blind expert echolocators [four EB (early-blind) and one LB (late-blind), all male, ages 16–58 years] participated. Proficient echolocators were recruited based on self-reports of long-term and active use of mouth-clicking echolocation for daily life activities (see details in Table 1). Sighted controls were untrained in echolocation and reported normal or corrected-to-normal vision. Both sighted and blind individuals were naive to the task; they had normal hearing as assessed with pure tone audiometry (250–8,000 Hz) and gave informed consent following our protocol approved by the Smith–Kettlewell Eye Research Institute Institutional Review Board.

**Table 1.** Blind expert echolocator participants

Blind individuals					
Subject ID	Blindness onset (year)	Age at test (year)	Blindness severity	Blindness etiology	Echolocation expertise (# years of practice)
EB1	0	46	Total	Glaucoma and optic nerve damage	>40
EB2	<1	57	Total	Retinoblastoma (Enucleation)	∼57
EB3	0	16	LP	Retinal detachment	11
LB1	14	39	Total	Optic nerve atrophy	24

LP, light perception.

### Stimuli

Stimuli consisted of a ∼6 ms convolved sound of a mouth click and its spatialized echoes reflecting a virtual object located 1 m away at various eccentricities in the azimuthal plane. To generate the stimuli, we first synthesized a mouth click using parameters and code from Thaler et al. (2017), modeling spectrotemporal waveform features and azimuthal directivity of clicks produced by skilled human echolocators (∼3 ms duration, power distributed from ∼1–15 kHz, peaking at ∼2–4 kHz). To simulate the spatial origin of the oral click, the waveform was spatialized at 0° (directly in the frontal plane) by convolution with a generic head-related transfer function (HRTF; Gardner and Martin, 1995). The echo was simulated by introducing a time delay corresponding to the 1 m distance (∼5.8 ms at 343 m/s) plus an estimated 12 cm between the mouth and ear, attenuation from geometric spreading loss, and the emitted pulse's azimuthal directivity and spatialization via HRTF convolution at 5, 10, 15, 20, or 25° to the left or right of center. No losses from target reflectivity were modeled. The final binaural stimulus, comprising mouth clicks and echoes, was ∼6 ms in duration.

### Task and experimental procedure

The experiment was conducted in a darkened, sound-damped testing booth (VocalBooth). Participants sat 57 cm from a 27″ display (Asus ROG Swift PG278QR, Asus), wearing the EEG cap with 64 channels and tubal-insert earphones (Etymotic ER-3A). Stimuli were binaural and presented dichotically. We used the MOTU UltraLite Mk4 Audio Interface (MOTU) to improve the temporal precision of auditory stimulus presentation and synchronization to EEG triggers via pass-through detection (StimTrak, Brain Products). Auditory stimuli were presented at 64 dB SPL.

**Figure 1.**
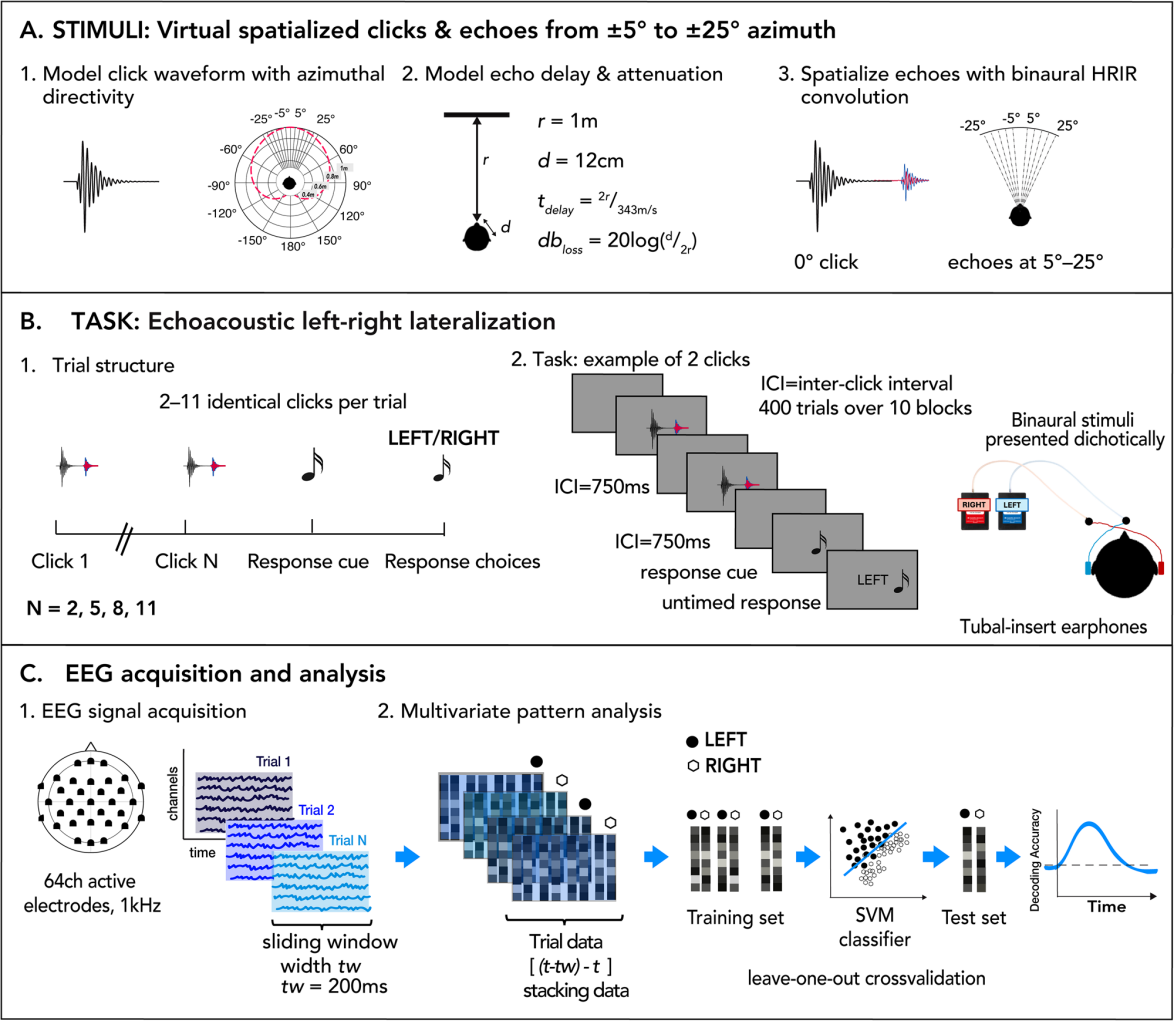
Stimulus generation, experimental design, and EEG acquisition/analysis. ***A***, STIMULI: (1) Click generation and modeling azimuthal directivity (Thaler et al., 2017). (2) Echo delay and attenuation modeled based on 1 m distance. No reflectivity loss was modeled. (3) Echo spatialization (5–25°) using head-related impulse response (HRIR; Gardner and Martin, 1995). ***B***, TASK: (1) Trials structured in trains of 2, 5, 8, or 11 clicks. (2) Task: an example of a trial of two clicks. ***C***, EEG acquisition and analysis: (1) EEG signal acquisition. (2) MVPA pipeline to generate decoding time courses.

**Figure 2.**
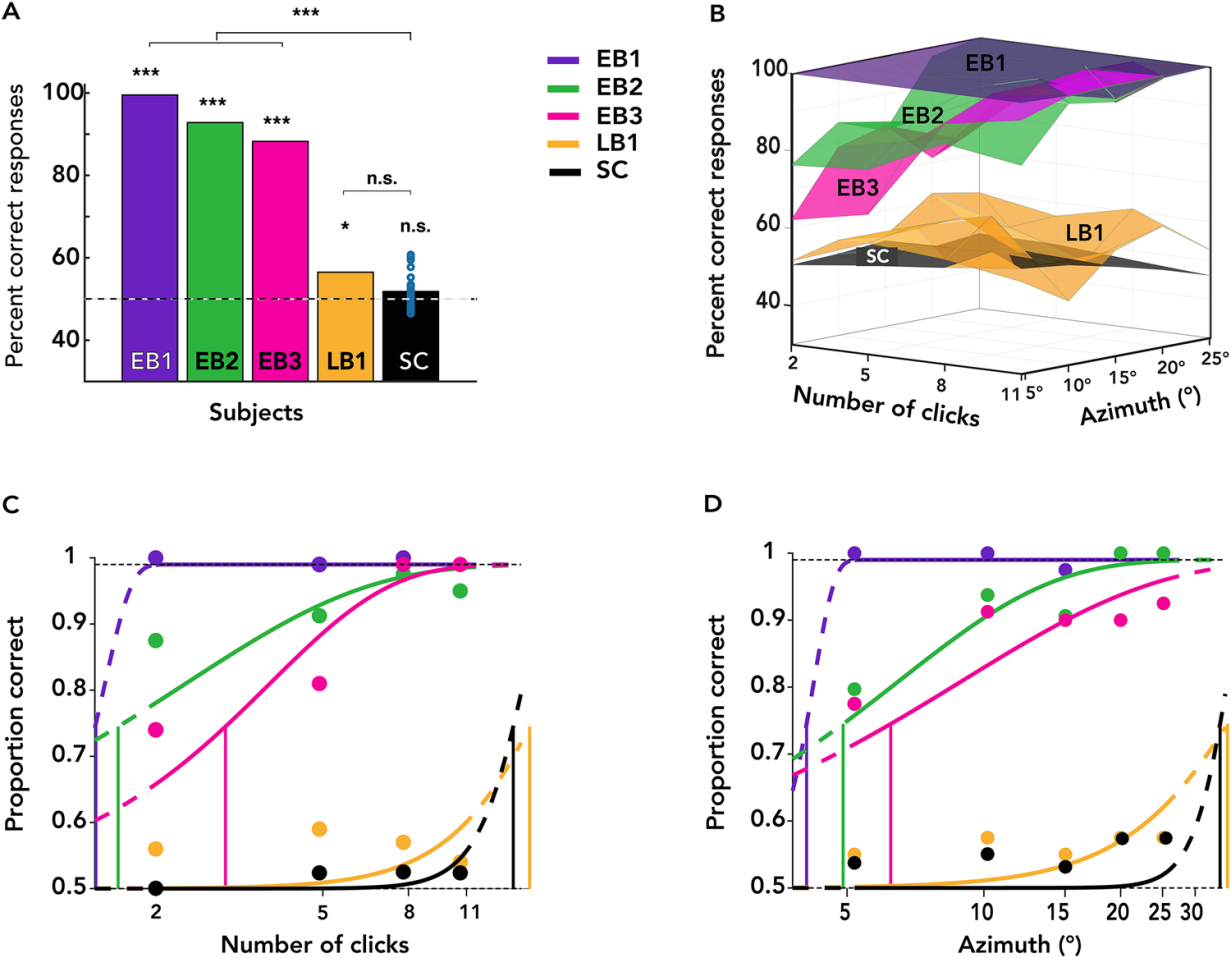
Task performance from EB, LB, and SC participants. ***A***, Individual task performance according to visual experience. ***B***, 3D visualization of task performance for azimuth and number of clicks. ***C***, ***D***, Psychometric functions for number of clicks and azimuth for each blind echolocator and the average of sighted controls (*N* = 21).

**Figure 3.**
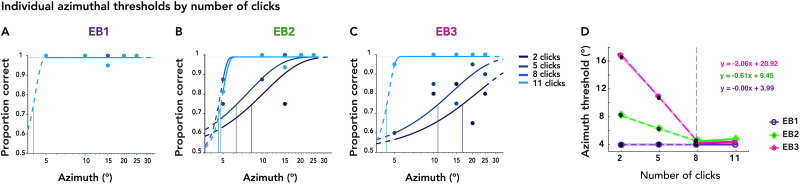
Individual psychometric functions for azimuth across click-echo conditions in EB echolocators. ***A–C***, Psychometric curves are shown for each EB participant, illustrating localization performance as a function of azimuth angle, separately for different numbers of clicks. Vertical lines indicate 75% thresholds. ***D***, Thresholds plotted against the number of click-echoes. Piecewise linear regression (dashed lines) is used to assess the change in spatial precision across the first three click-count conditions before the ceiling-performance plateau.

Participants completed a 2AFC auditory localization task in which they reported whether a virtual object was located to the left or right relative to the midsagittal plane. For sighted participants, each trial began with a white fixation cross against a gray display, followed by a sequence of 2, 5, 8, or 11 identical click-echo stimuli, separated by a stimulus-onset asynchrony of 750 ms (Fig. 1*B*-2). The number of clicks was not indicated ahead of time. Following the final stimulus in the train, a 5 ms 180 Hz tone cued an untimed response display; participants responded in two steps: cycling through “left” and “right” response options using the up and down arrow keys and then confirming with either of the adjacent (left/right arrow) keys. The initially displayed response option was randomized. This response scheme was made clear to subjects from the start of the experiment during the practice blocks. By jittering the sequence of keypresses needed for a given response in each trial, we decoupled stimulus-related processing from motor response planning and execution, as in previous work (García-Lázaro and Teng, 2024). A 5 ms 200 Hz tone confirmed the response and initiated a jittered intertrial interval of 700–1,250 ms before the next trial (Fig. 1*B*-3).

We blocked trials into 10 groups of 40 trials each, presented in a random order and corresponding to 40 conditions created by combining 10 eccentricities (±5, ±10, ±15, ±20, and ±25°) and 4 sequences of varying clicks (2, 5, 8, and 11). We collapsed left and right positions to focus on absolute eccentricity relative to the midline. Each block lasted ∼7 min, with breaks between blocks as needed, for a total experiment time of ∼70–75 min. We used Psychtoolbox-3 (Pelli, 1997) running in MATLAB 2021b (The MathWorks) for stimulus presentation, the MATLAB Audio Processing Toolbox, and the voice changer tool (*Time-Domain Pitch and Time Scale Modification of Speech Signal*) to speed spoken instructions for blind individuals (1.8 times the normal speed; Nguyen and Nguyen, 2008).

### EEG data acquisition and preprocessing

We recorded continuous EEG using a Brain Products actiCHamp Plus recording system (Brain Products) with 64 channels arranged in a modified 10–20 configuration on the caps (Easycap). The *Fz* channel was used as an online reference during the recording. We used the StimTrak device (Brain Products GmbH) as an auxiliary EEG channel connected to amplifiers and audio output earphones to tag stimulus-onset times more precisely. The EEG signal was bandpass filtered online from 0.01 to 500 Hz and digitized at 1,000 Hz. The continuous EEG signal was preprocessed offline using the FieldTrip toolbox (Oostenveld et al., 2011) and customized scripts using MATLAB functions for downsampling and filtering the neural signal. Raw data were rereferenced to the common average of all electrodes and segmented into epochs of different lengths according to conditions. Stimulus-onset times were corrected and aligned with the timestamps provided by StimTrak to increase time precision. Trials of the first two and last two clicks were segmented from −400 to 1,400 ms relative to the stimulus onset of interest. Trials of 5 clicks were segmented from −400 to 3,700 ms; trials of 8 clicks were from −400 to 5,950 ms; and trials of 11 clicks were from −400 to 8,200 ms. Epochs were baseline corrected, downsampled by averaging across nonoverlapping 10 ms windows (Guggenmos et al., 2018), and low-pass filtered at 30 Hz. Trials were labeled by target laterality (right, left) and number of clicks (2, 5, 8, or 11).

### EEG multivariate pattern analysis (MVPA)

We analyzed the EEG signal using linear support vector machine (SVM) classifiers to decode neural response patterns at each time point of the preprocessed epoch using a MVPA approach (Fig. 1*C*). We applied a retrospective sliding window in which the classifier for time point *t* was trained with preprocessed and subsampled sensor-wise data in the interval [*t*-20, *t*]. This method increases the SNR and captures the temporal integration of dynamic and nonstationary properties (García-Lázaro and Teng, 2024). The resultant decoding time course thus began at −200 ms relative to stimulus onset. Pattern vectors in the window were stacked to make a composite vector. For example, 21 samples of 64-channel data made a composite vector that was 1,344 elements long. Decoding was conducted using custom MATLAB scripts that adapted functions from Brainstorm's MVPA package (Tadel et al., 2011) and *libsvm* (Chang and Lin, 2011). We used 10-fold cross-validation (leave-one-fold-out), whereby trials from each class were randomly partitioned into 10 subsets and averaged within each subset (Guggenmos et al., 2018). In each iteration, one fold-average served as the test sample while the classifier was trained on the remaining nine. This procedure was repeated across 100 random fold assignments, and decoding accuracy at time *t* was calculated as the mean across permutations.

### Statistical testing

To assess the statistical significance of behavioral indices, we used a one-sample binomial test to contrast the proportion of correct responses against chance (50%) for single-blind individuals and a *t* test for the group of sighted controls. To compare the performance of blind echolocators with sighted controls, we used an adjusted one-tailed *t* test analysis for small-sample sizes as described by Crawford et al. (2010) and Crawford and Howell (1998). The statistical significance of the EEG decoding time courses across sighted subjects was determined using *t* tests against the null hypothesis of chance level (50%). We used nonparametric permutation-based cluster–size inference to control for error rate inflation in multiple comparisons. The cluster threshold was set to *α* = 0.05 (right-tailed) with 1,000 permutations to create an empirical null hypothesis distribution. The permutation distribution's significance probabilities and critical values were estimated using a Monte Carlo simulation (Maris and Oostenveld, 2007). The statistical significance of the EEG decoding time courses for single subjects was determined by contrasting them against a null distribution generated by randomly shuffling labels 100 times under the null hypothesis that left versus right labels were interchangeable. Decoding accuracy of the observed data was significant if it exceeded 95% of the permuted decoding scores.

### Modeling EEG signatures of decision readout policies

Performance improvements across repeated click-echo samples could reflect cumulative evidence integration but may also arise from alternative computational readout rules, such as reliance on a single optimally encoded sample. To distinguish these accounts at the level of decision readout, we fit trial-level robust logistic regression models predicting echo lateralization accuracy (correct/incorrect) from behavioral and EEG-derived predictors. These robust models incorporated a Jeffreys prior to reduce small-sample bias and a lapse-mixture component, allowing a bounded lapse rate (maximum, 0.20) to account for occasional responses not driven by stimulus evidence. No L2 regularization was applied. These options improve parameter stability when trial counts are limited or performance approaches ceiling.

For each click within a trial, we extracted a scalar EEG metric, defined as the mean absolute amplitude from 50 to 200 ms following click onset (Ungan et al., 2001; Edmonds and Krumbholz, 2014). This yielded a sequence of click-wise EEG response magnitudes per trial, analogous to trial-level neural decision variables used in prior studies of perceptual decision-making (Murphy et al., 2015). From these click-wise sequences, we constructed four trial-level EEG predictors reflecting distinct candidate readout policies: two integration-consistent readout rules that differ in how click-wise EEG responses are weighted (*SUM* and *SLOPE*), one nonintegrative readout rule based on the best sample (*MAX*), and one hybrid readout rule that interpolates between integration and single-sample selection (*SOFTMAX*).

*Cumulative evidence (SUM)* was defined as the total sum of the magnitude of click-wise EEG responses within a trial, implementing an accumulation-consistent readout in which each click contributes additively to the decision variable. *SLOPE* was computed as the linear slope of click-wise EEG response magnitude as a function of click order within each trial. The slope quantifies systematic variations in the EEG response magnitude across the click sequence (Hyafil et al., 2023). *Single best-sample evidence (MAX)* was defined as the maximum click-wise evidence value within a trial, representing a nonintegrative readout rule in which performance could improve with repetition because additional clicks increase the probability of obtaining one strongly encoded sample (Stine et al., 2020; Hyafil et al., 2023). Finally, *Softmax pooling (SOFTMAX)* combines click-wise evidence using graded weights, such that all clicks contribute to the decision. Their influence depends on their relative strength and the sensitivity parameter *β*, which controls how strongly evidence differences shape the final weighting (Pisupati et al., 2021; Fritsche et al., 2024). For primary analyses, the parameter *β* was fixed at 3, representing an intermediate weighting regime. Robustness was evaluated by repeating the analysis across *β* values ranging from 0 to 8 in steps of 0.5, spanning near-uniform weighting (low *β*) to highly selective weighting (high *β*). To isolate trial-by-trial neural contributions beyond the effect of sequence length, all EEG-derived predictors were residualized with respect to the number of clicks (log-transformed) using linear regression prior to model fitting. Residualization reduces the influence of variance linearly associated with sequence length, minimizing trivial performance gains driven solely by longer sequences and allowing model comparisons to reflect trial-to-trial neural variability beyond repetition-related effects.

#### Model specification and comparison

We first fit a baseline behavioral model that included only the number of clicks as a predictor of accuracy. This model served as a reference, quantifying how much of the performance improvement was attributable to repetition alone. Each EEG-augmented model then added one residualized EEG predictor (*SUM*, *MAX*, *SOFTMAX*, or *SLOPE*) to the baseline model. Models were compared using corrected Akaike information criterion (AICc) and 10-fold cross-validated predictive performance. Generalization was quantified relative to the baseline model on held-out folds using changes in area under the ROC curve (ΔAUC) and changes in root mean squared error (ΔRMSE). Inference about the best-supported decision readout policy relied primarily on cross-validated metrics, with information criteria used as converging evidence. This framework compares alternative ways of combining sequential neural samples without imposing a specific generative accumulation model. For SOFTMAX models, *β* was examined descriptively to characterize the relative weighting of clicks within the model (i.e., whether behavior approached additive integration or winner-take-all selection).

These models compare alternative readout policies for combining sequential neural samples without imposing assumptions about the underlying generative mechanism.

## Results

### Expert blind echolocators outperformed sighted controls in echolateralization

Overall performance for each echolocator and the group of sighted controls is shown in Figure 2*A*. Blind echolocators were not all equally accurate, but all of them were better than chance at lateralizing the echoes (one-sample binomial test: EB1 mean = 99.5, *p* < 0.001; EB2 mean = 92.81, *p* < 0.001; EB3 mean = 88.25, *p* < 0.001; and LB1 mean = 56.5, *p* < 0.05; chance = 50). In contrast, sighted controls, as a group, performed at chance [sighted control (SC) mean = 51.83; SEM = 0.98; *t*_(20)_ = 1.86; *p* = 0.076]. EBs’ performance compared with SC's performance using an adjusted one-tailed *t* test analysis for small samples (Crawford and Howell, 1998; Crawford et al., 2010) revealed that EBs, on average, differed from sighted controls (EBs’ mean = 93.52; SC mean = 51.83; SD = 4.50; *t* = 8.04; *p* < 0.001). LB performance did not statistically differ from SC performance (*t* = 1.04; *p* = 0.16).

Figure 2*B* plots a 3D visualization of condition-wise performance for each individual and group, with the *x*-axis indicating click counts from 2 to 11 and the *y*-axis indicating absolute azimuthal eccentricities from 5 to 25°. We observed individual differences in echolocation performance between experts; for example, EB1 (violet) performed near ceiling in the task, while LB1 did not exceed 75% in any condition. In contrast, sighted controls did not perform higher than 61% when split by the number of clicks or eccentricities.

### Performance improved with azimuth and clicks, varying by expertise

To further investigate the role of click count and azimuthal eccentricity in echolateralization, we computed psychophysical thresholds for each blind participant and pooled sighted controls across all trials, grouping scores by the number of clicks or azimuth angles. The underlying psychometric curve was modeled using the Weibull function (Teng et al., 2012), with thresholds defined as the stimulus eccentricity or click count at which participants correctly lateralized echoes 75% of the time. The lapse rate was fixed at 0.01, with slope, width, and threshold set as free parameters. We used the psignifit4 toolbox (Schütt et al., 2016) in MATLAB 2021b for these analyses.

Figure 2, *C* and *D*, displays separate psychometric functions for each EB and LB participant, along with a combined psychometric fit for SC, for azimuth and number of clicks. For EB participants, the proportion of correct responses improved with both increased azimuth degrees and the number of clicks. Thresholds for azimuth and number of clicks, respectively, varied across participants: EB1 (3.89°; 1.42 clicks; violet), EB2 (4.71°; 1.62; green), and EB3 (6.03°; 2.95; pink). In contrast, LB's and SC's chance or near-chance performance resulted in thresholds beyond the range of the sampled stimulus space for both azimuth and number of clicks: LB1 (34.88, 16.21; yellow) and SC (33.57, 14.80; black). As a result, subsequent analyses focus on the three EB participants.

### Azimuthal thresholds decreased with additional clicks in EB echolocators

Next, to assess the effect of repeated clicks on localization performance, we computed separate azimuthal thresholds for trials grouped by click count. Figure 3*A–C* shows individual psychometric fits for EB1, EB2, and EB3 for trials with 2, 5, 8, and 11 clicks, indicated by gradually lighter shades of blue. Individual thresholds by click count are plotted in Figure 3*D*. For EB2 and EB3, azimuthal thresholds decreased with increasing number of clicks, reaching a plateau after eight clicks for EB2 and EB3. In contrast, EB1's ceiling-level thresholds were consistent regardless of the number of clicks. This suggests that EB2 and EB3 benefited from repeated identical click presentations, while EB1 needed no more than two clicks to perfectly lateralize echoes within a range of 5–25°. EB1's ceiling-level thresholds also serve as a benchmark for performance limits in this experiment, indicating that EB2 and EB3 thresholds plateaued after eight clicks.

To quantify performance improvements with repeated clicks, we fitted piecewise linear models to the first three data points for each participant to capture the initial change rate before the plateau. The degree of improvement varied across individuals, reflected both in the initial threshold with two clicks and the slope of improvement with additional clicks, as captured by the model's parameters. Figure 3*D* illustrates the linear fit for EB2: the model predicted an azimuthal precision of ∼8° with two clicks, with each additional click improving precision by ∼0.61° (threshold = −0.61 × clicks + 9.45). For EB3, the model estimated a higher starting threshold of ∼21°, with a steeper improvement of ∼2° per additional click (threshold = −2.06 × clicks + 20.42). EB1 already exhibited low thresholds with two clicks (∼3.99°), with no change at repetition counts.

Together, these results highlight a progressive improvement in spatial precision as the number of clicks increases, quantifying the estimated rate of information gain for each individual.

### Effect of azimuth on click-based thresholds for object spatial location

**Figure 4.**
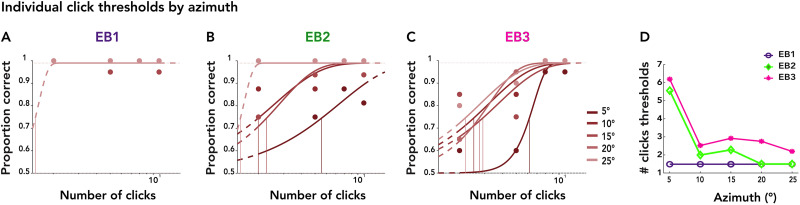
Individual psychometric functions for each EB echolocator, for the number of clicks as the stimulus level, grouped by azimuth. ***A–C***, Psychometric curves illustrate localization performance as a function of the number of clicks, plotted separately for each azimuth condition (red). ***D***, Thresholds (derived from psychometric functions) are plotted against azimuth.

We next examined how azimuthal eccentricities related to the number of clicks required for accurate spatial localization. We grouped trials by the azimuth angle to fit individual psychometric functions for the number of clicks. Figure 4 displays these psychometric functions for EB1, EB2, and EB3 with azimuths ranging from 5° (dark red) to 25° (light red) in increments of 5°. The threshold patterns shown in Figure 4*A–C* reveal distinct individual differences across azimuths. For objects located at 5° of eccentricity, EB2 and EB3 required ∼5.5–6.5 clicks to localize accurately. Thresholds declined sharply from 5 to 10° and then more gradually from 10 to 25°, suggesting that beyond 10° of eccentricity, EB2 and EB3 needed 1.5–3 clicks to localize objects. Notably, EB1 thresholds quickly reached 1.5 and remained steady at it across all azimuths, indicating that two clicks provided sufficient information to localize objects from the entire 5–25° range. These findings reveal individual differences in the number of clicks required to achieve spatial precision and illustrate that laterality information varies with eccentricity: a less eccentric echo has “less” laterality information that may require more clicks to perceive.

### Neural representations of spatial object laterality across early and late clicks

Next, we examined how echoacoustic spatial information is represented in neural activity by applying MVPA to EEG data. To capture the temporal evolution of spatial representations, we classified neural responses between left- and right-lateralized echoes, focusing on two time windows within each trial: the first two clicks from all trials and the final two clicks from sequences of 5, 8, and 11 clicks. We restricted decoding analysis to the dataset of 5-, 8-, and 11-click trials to ensure that late-phase effects could not be trivially explained by early-phase activity in the 2-click condition (where the first two and last two clicks are identical). This allowed us to assess neural representations just after trial onset and just before the response while analyzing completely nonoverlapping data. Importantly, as physical stimulation, participant sample, trial count, etc. were identical across these windows, differences in decoding dynamics are therefore more consistent with sequence-position–dependent changes in neural state than with differences in physical stimulation.

#### Neural spatial encoding during early clicks

**Figure 5.**
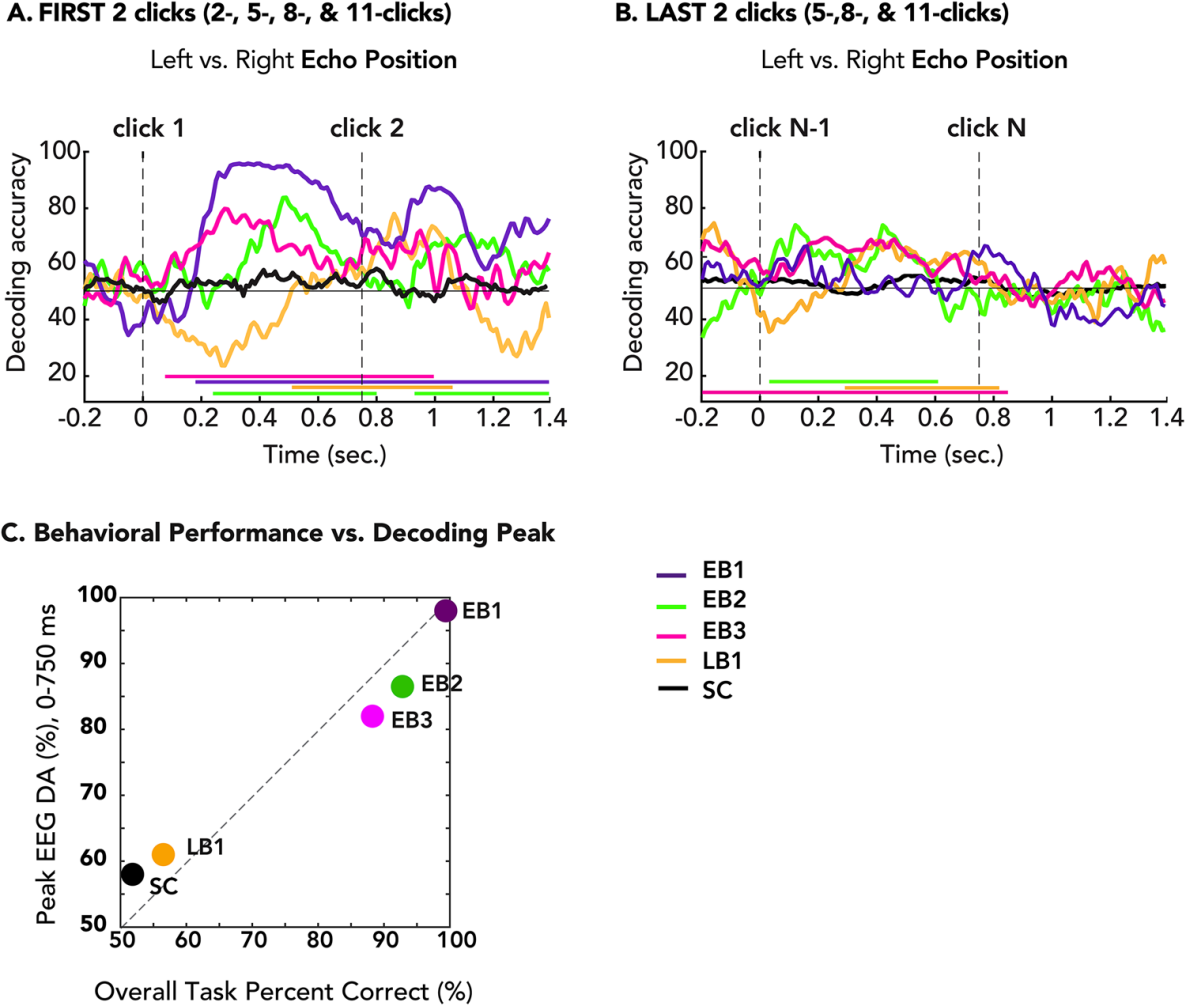
Decoding time courses for echo laterality following the first two clicks from all trials (***A***), and the last two clicks from 5, 8, and 11 clicks trials (***B***). Vertical dashed lines indicate click onsets at 0 and 750 ms. Horizontal dashed line indicates chance level (50%), and colored bars along the *x*-axis denote significance. For SC, group-level statistics were computed (*N* = 21) using a *t* test against chance (50%), with a cluster-defining threshold of *p* < 0.05 and 1,000 permutations. For each blind individual, single-subject statistics were computed by comparing each participant's decoding curve to an empirical null distribution generated by applying SVM decoding to label-randomized trials, permuted 100 times. ***C***, Correlation between the first DA peak from all trials in the 0–750 ms window (*y*-axis) and overall task performance (*x*-axis). EB participants (EB1–3) exhibit both higher decoding peaks and greater behavioral accuracy, whereas the LB participant (LB1) and the sighted control (SC) show lower values. This pattern indicates a relationship between early spatial neural decoding and task performance.

Figure 5*A* shows decoding time courses for individual participants and the pooled SC group, color-coded as in Figure 2. In EB1 (violet), decoding accuracy rose sharply ∼180 ms after the first click, peaked at 98%, dipped slightly ∼600 ms, and then rose again following the second click, remaining elevated through the end of the epoch. Similarly, EB2 (green) exhibited above-chance decoding from 240 to 800 ms and 930 to 1,400 ms, while EB3 (pink) demonstrated significant decoding from 70 to 990 ms. All EB participants exhibited robust and sustained decoding accuracy following both clicks, with peak decoding accuracy consistently occurring after the first click and a secondary peak following the second click. In contrast, LB1 (orange) showed above-chance decoding from 510 to 1,060 ms, with peak accuracy following the second click. Sighted controls (black) showed no significant decoding throughout the epoch.

#### Neural response of spatial encoding during final clicks

Next, we analyzed neural activity during the last two clicks (5, 8, and 11 click sequences). As shown in Figure 5*B*, EB1 (violet) did not reach significance. EB2 (green) showed above-chance decoding across two intervals from ∼50 to 600 ms and 1,000 to 1,300 ms, whereas EB3 (pink) exhibited a sustained significant period from −200 to 600 ms. In contrast, LB1 (orange) and sighted controls (black) did not exhibit above-chance decoding throughout the final two clicks.

#### Relationship between decoding and behavior

To relate neural discriminability to behavior, we plotted each participant's first decoding peak decoding accuracy (0–750 ms) against overall task accuracy (Fig. 5*C*). EB participants clustered toward higher values on both axes, whereas LB1 and SC showed lower decoding and lower performance. This correspondence suggests that stronger neural discriminability in early clicks was associated with higher overall task accuracy across participants.

### Neural representations evolve across click-ordinal positions within a trial

**Figure 6.**
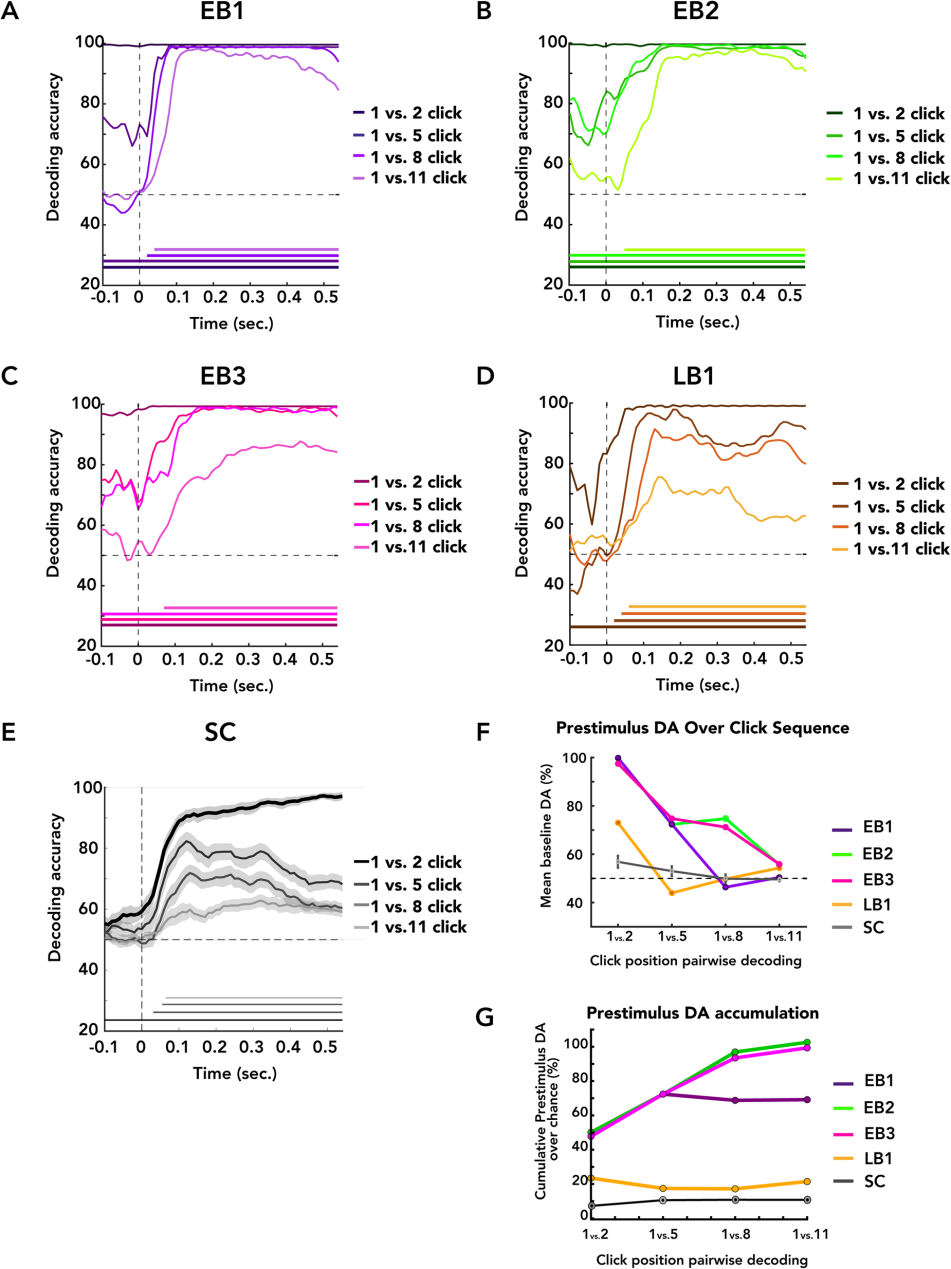
Neural decoding across click sequences. ***A–D***, Decoding accuracy time courses for individual blind echolocators. Colored lines indicate SVM classification contrasting the first click with later clicks within each sequence (1 vs 2, 1 vs 5, 1 vs 8, 1 vs 11). Each click was analyzed as a single-click epoch from −200 to 550 ms relative to click onset. Horizontal bars along the *x*-axis mark time windows of significant above-chance DA (null permutation; cluster-corrected, *p* < 0.05). ***E***, Decoding accuracy time courses for the pooled SC group using the same comparisons as blind participants. Group-level statistics were computed (*N* = 21) using a *t* test against chance (50%), with a cluster-defining threshold of *p* < 0.05 and 1,000 permutations. ***F***, Mean decoding accuracy during the baseline period (−200 to 0 ms) and click comparison (1 vs 2, 1 vs 5, 1 vs 8, 1 vs 11) for each participant. ***G***, Cumulative sum of decoding accuracy across comparisons relative to chance. Larger slopes indicate greater differentiation between sequence-position neural dynamics, whereas flatter profiles indicate smaller discriminability across click-ordinal positions.

We next examined whether neural responses varied across click-ordinal positions within a trial by testing how discriminative patterns changed as a function of the click number. Observed across the click sequence might be accompanied by systematic changes in brain responses beyond those expected from identical stimulus repetitions alone. To preserve statistical power, we focused on comparisons between the first click and later clicks within each sequence (1 vs 2, 1 vs 5, 1 vs 8, and 1 vs 11). Each click was analyzed using a −200 to 550 ms window relative to click onset. Between-ordinal-position click decoding results are shown for each blind echolocator in Figure 6*A–D* and for SC in Figure 6*E*. A consistent pattern was observed: Click 1 was robustly distinguishable from Click 2 across each epoch and progressively less so for Clicks 5, 8, and 11. Across participants, postclick responses for Clicks 2, 5, 8, and 11 became distinguishable from Click 1 within ∼100 ms and remained so for the rest of the epoch. Sighted controls exhibited a qualitatively similar trend but were markedly weaker and less persistent effects, suggesting that sequence-position–dependent neural differentiation is present in sighted listeners but reduced relative to expert echolocators. This general pattern was evident both in postclick neural responses and in the interval preceding click onset (preclick). We further focused on the preclick interval in this analysis as a reasonable candidate window for identifying persistent or sequence-dependent signal changes less likely to be dominated by click-evoked sensory responses. The preclick interval was progressively less discriminable from (i.e., more similar to) the true prestimulus baseline for later clicks relative to the first (Fig. 6*F*). For example, EB1's Click 1 baseline was highly distinguishable from Click 2, ∼70% decodable from Click 5, and not significantly distinguishable from Clicks 8 and 11. EB2 and EB3 showed baseline discriminability until Click 11, whereas for LB1 and SC, only Click 2 differed from Click 1. Finally, preclick discriminability versus Click 1 was operationalized as decoding accuracy above-chance level, i.e., DA from Figure 6*F* minus 50%, and progressively summed over the click sequence. LB1 and SC showed weak or no cumulative change, while EB1–3 exhibited initial large gains that leveled off following Clicks 5 and 8 (Fig. 6*G*). This graded structure broadly mirrors changes in behavioral performance across the click sequence.

**Figure 7.**
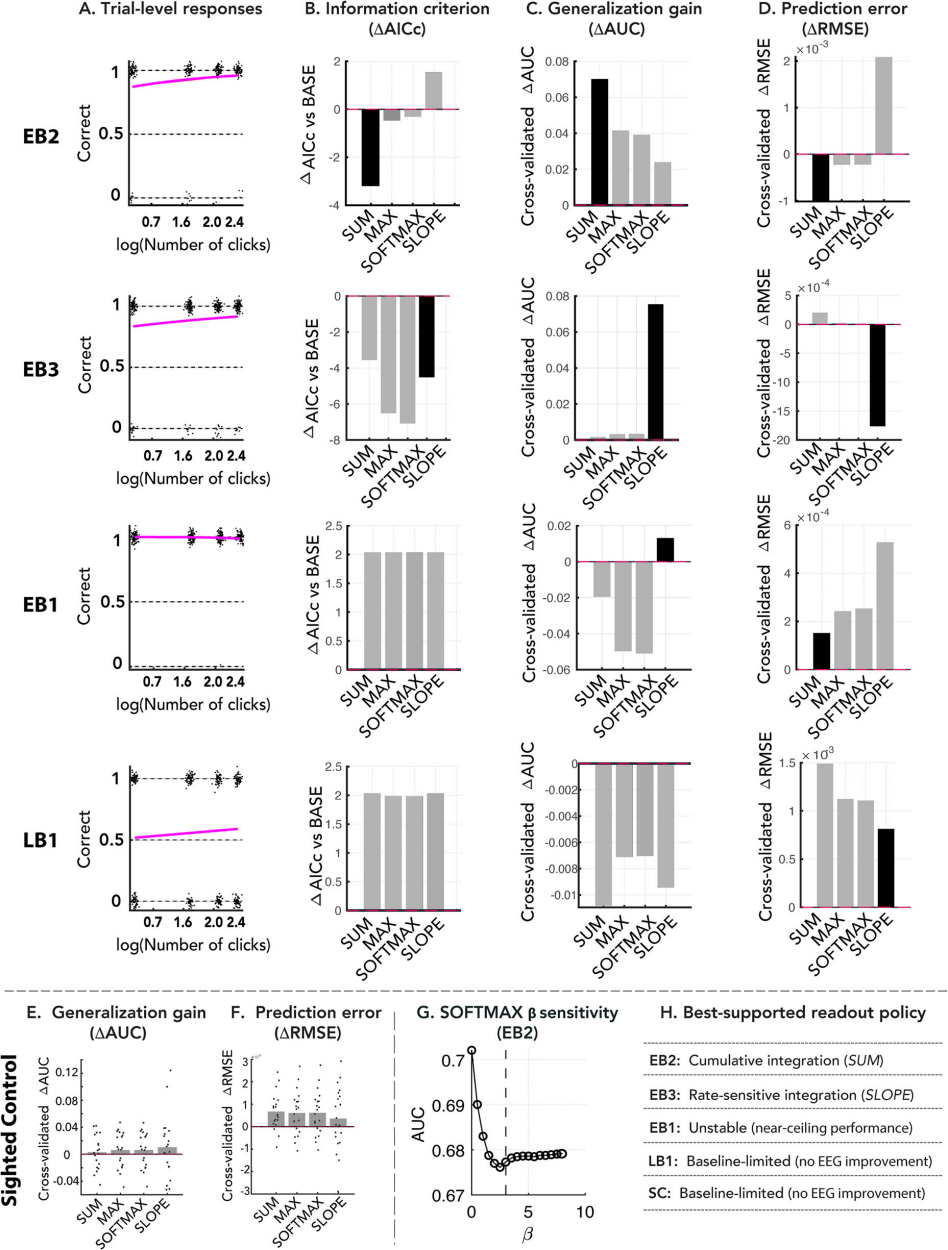
Trial-level model comparison of EEG-based candidate decision readout policies for each blind echolocator (four top rows) and pooled across sighted controls. ***A***, Trial-level behavioral performance as a function of the number of clicks (log-transformed) for each blind participant. Points indicate individual trials; solid lines show the fitted baseline behavioral model. ***B***, Information criterion differences (ΔAICc) relative to the baseline model, reflecting relative in-sample model fit. Lower values indicate better model fit. ***C***, Generalization gain quantified as cross-validated ΔAUC relative to the baseline model. Positive values indicate improved out-of-sample discrimination. ***D***, Prediction error change quantified as cross-validated ΔRMSE relative to the baseline model. Negative values indicate reduced prediction error. ***E***, ***F***, Group-level summary across SC (*N* = 21). ***E***, Cross-validated generalization gain (ΔAUC). ***F***, Cross-validated prediction error change (ΔRMSE). Error bars indicate group means; dots indicate individual participants. ***G***, Softmax *β*-sensitivity for representative participant EB2. Cross-validated performance varies smoothly across *β*, indicating that the softmax model spans a continuum from uniform averaging to max-like selection. The dashed line indicates the value used in the main analyses (*β* = 3). ***H***, Decision readout profile summary indicating the decision readout pattern most supported for each participant based on converging evidence from cross-validated performance, prediction error, and information criterion metrics.

These results show that neural responses differed systematically across sequence positions, with early clicks more distinguishable than later clicks and baseline activity becoming progressively more similar across later positions. The functional relevance of these neural dynamics is evaluated explicitly in the subsequent trial-level model comparison analyses (Fig. 7).

### Model comparison favors accumulation-consistent readout policies over single-sample alternatives

The analyses above establish that performance improves with repeated click-echo samples (when observers can perceive them at all) and that neural representations of laterality evolve systematically across the click sequence. Our trial-level model comparisons using EEG-derived click-wise evidence metrics aimed to determine whether these trends are more consistent with integrative (*SUM* and *SLOPE*), nonintegrative (*MAX*), or hybrid (*SOFTMAX*) accounts of echoacoustic information processing.

Across blind participants, model comparisons revealed distinct individual profiles. For EB2, adding cumulative neural evidence (SUM) produced the largest improvement relative to baseline across metrics (ΔAUC ≈ +0.07; lowest ΔAICc; lowest ΔRMSE), whereas MAX, SOFTMAX, and SLOPE provided smaller gains. Thus, EB2's performance was best accounted for by a cumulative readout of sequential neural evidence (Fig. 7*A*–*D*, Row 1). For EB3, the SLOPE predictor yielded the strongest improvement in cross-validated discrimination and prediction error reduction, outperforming all other models and producing the lowest ΔAICc (Fig. 7*A*–*D*, Row 2). For EB1, performance was near ceiling across conditions, resulting in minimal trial-level variance and unstable parameter estimates. Consequently, EEG–behavior relationships for this participant are reported only descriptively rather than inferentially (Fig. 7*A*–*D*, Row 3). By comparison, for LB1 individually, none of the EEG-augmented models improved model performance relative to baseline (all ΔAUC ≈ 0 or negative; ΔAICc > 0; ΔRMSE ≥ baseline), indicating no reliable trial-level contribution of EEG-derived predictors under the tested readout rules (Fig. 7*A*–*D*, Row 4). At the group level for sighted controls (SC; *N* = 21), EEG-augmented models did not reliably improve generalization performance, with ΔAUC values centered near zero and minimal changes in prediction error across readout rules (Fig. 7*E*,*F*). Thus, neural predictors did not systematically account for trial-wise performance variability in sighted listeners.

To verify that softmax pooling behaves as an intermediate readout rule, we evaluated performance across *β* values. For representative participant EB2, cross-validated performance varied smoothly as a function of *β*, consistent with the softmax model spanning a continuum between uniform averaging and max-like selection (Fig. 7*G*). Across participants, favored models differed (Fig. 7*H*), indicating heterogeneity in how neural evidence contributes to decisions. Nevertheless, no blind participant showed evidence favoring a strict single-sample readout over accumulation-consistent alternatives when behavioral variability allowed reliable comparison. Together, these results indicate that performance is more consistent with readout mechanisms that incorporate information across clicks than with reliance on isolated samples while still revealing marked individual differences in how sequential evidence is weighted.

## Discussion

In this study, we investigated the neural and behavioral mechanisms underlying echoacoustic spatial perception by combining psychophysics with EEG recordings in blind expert echolocators and sighted novice controls. Participants performed a spatial lateralization task using virtual echoes derived from synthetic clicks, allowing us to examine how temporally distributed auditory cues contribute to spatial representations. Expert blind echolocators exhibited significantly greater accuracy than novice sighted controls, suggesting experience-dependent differences in spatial processing. Localization accuracy improved systematically with both eccentricity and the number of echoacoustic samples. Neural decoding analyses closely mirrored behavioral performance, suggesting that dynamic neural representations reflected the evolving spatial perception. Trial-level model comparisons further indicated that performance was more consistent with integration of neural evidence across clicks than with reliance on single-sample alternatives. These findings suggest that perceptual and neural dynamics of spatial representations in echolocation are consistent with construction through progressive integration.

### Expert blind echolocators exhibit superior spatial localization, shaped by visual experience

Consistent with prior research findings (Rice, 1967, 1969; Vercillo et al., 2015, 2017; Wallmeier et al., 2015), blind expert echolocators outperform sighted controls in localizing objects. Although all blind echolocators performed above chance, EB individuals were more accurate, performing at >70% even with only two clicks. LB's lower performance comes despite being proficient in real-world echolocation and serving as an echolocation instructor. This performance difference based on blindness onset aligns with findings by Thaler et al. (2011), who reported lower performance in an LB expert compared with EB, including chance-level lateralization of passively presented click-echo recordings. More broadly, the behavioral results are consistent with the idea that blindness onset may critically shape spatial hearing. Early blindness may foster auditory-based spatial priors and enhanced sensitivity to binaural cues (Röder et al., 1999; Nilsson and Schenkman, 2016), while later blindness leaves individuals relying on visually calibrated reference frames less suited to auditory judgments such as echolocation (Amadeo et al., 2019). This aligns with broader evidence that spatial perception is visually calibrated (Eimer, 2004; Röder et al., 2008; Gori et al., 2014; Sourav et al., 2018; Röder and Kekunnaya, 2021) and suggests that early deprivation coupled with echolocation enhances sensitivity to spatial acoustic cues and supports more efficient neural mechanisms for spatial decisions (Vercillo et al., 2017).

### Psychophysical thresholds reveal temporal evidence accumulation and spatial cue salience effects

Localization performance improved with increasing azimuthal eccentricity and additional clicks, especially in EB participants. These findings are consistent with an evidence-accumulation account in which spatial information is scaled by eccentricity and integrated over repeated samples to drive perceptual decisions. The effect of azimuth likely reflects greater cue discriminability and enhanced interaural differences, supporting the notion that cue strength and salience jointly modulate the rate of evidence accumulation. This is corroborated by the click-count thresholds in Figure 4, showing that fewer clicks were needed to reach threshold performance when the echoes were more eccentric. We note that click acoustic energy is nearly isotropic within the range of our azimuths (Thaler et al., 2017) but that diminishing emission energy may offset the benefits of interaural cues at greater eccentricities. Recent work suggests an acoustically optimal eccentricity for echolocalization of ∼45° (Thaler et al., 2022).

From a signal detection perspective, multiple echoes increase the effective SNR available to the perceptual system by providing convergent information that boosts perceptual certainty (Thaler et al., 2018). Behaviorally, this process was captured by progressively decreasing lateralization thresholds with successive clicks in blind echolocators (Fig. 2). This pattern was clearest in EB2 and EB3, who showed steep improvements between the first and eighth clicks, suggesting their perceptual system effectively integrates echoacoustic features over time and then plateaus or saturates as ceiling performance is reached (EB1's thresholds did not improve with more clicks, as they were at ceiling level for all conditions). The approximately linear progression of thresholds provides a quantitative estimate of each individual click's contribution to precision: ∼0.6° per click for EB2 and 2° per click for EB3 (Fig. 3*D*). It also tentatively predicts a single-click lateralization threshold—8.8° for EB2 and 18.9° for EB3. Figure 2, *B* and *C*, also show that in addition to the threshold changes, EB2's and EB3's psychometric functions narrowed with more clicks, consistent with reduced uncertainty underlying the perceptual decision (Wichmann and Hill, 2001). LB1 performed above chance, but neither he nor sighted novices improved reliably across azimuths and click repetitions, suggesting that they were unable to extract and integrate meaningful spatial information from echoacoustic cues. Both LB1 individually and SC generally have been shown to echolocate successfully in other contexts (Teng and Whitney, 2011; Thaler et al., 2011; Norman et al., 2021, 2024), so some of the performance differences we show may be partly attributable to our experimental setup, e.g., the passive stimulus presentation and isolation of echoacoustics from other environmental cues. We also note that our task probed finer spatial grain (5–25°) than some previous studies by Rice (0–90°; Rice, 1967, 1969), Thaler et al. (0–180°; Thaler et al., 2018), and Wallmeier et al. (30–60°; Wallmeier et al., 2015).

Echoacoustic information can accumulate dynamically in multiple ways, e.g., through the greater SNR of louder clicks (Thaler et al., 2018, 2019), integrating across viewpoints for shape (Milne et al., 2014; Teng et al., 2024), or integrating across positions for moving objects (Thaler et al., 2013; Salles et al., 2020). Here, by synthesizing the echoacoustic stimuli, we kept acoustic stimulation and target–observer relationship constant across clicks and participants. Our psychophysical results thus suggest that variation in click- and azimuth-dependent improvement across individuals reflects differences in baseline abilities or processing strategies, rather than fluctuating sensory input or idiosyncratic strategies. The behavioral improvements with more clicks are also more consistent with an information-accumulation model of perceptual decision-making, where each echo contributes discrete evidence toward resolving spatial uncertainty, rather than a foraging-like search for a single optimal sensory signal.

### Neural signals represent spatial location early and dynamically in blind echolocators

While numerous studies have investigated the brain mechanisms of echolocation via fMRI (Thaler et al., 2011; Wallmeier et al., 2015; Flanagin et al., 2017; Norman and Thaler, 2019; Norman et al., 2024), to our knowledge, no prior EEG study has examined click-to-click neural dynamics during echoacoustic perception. EEG decoding timecourses revealed robust early neural discrimination of echo laterality in blind expert echolocators. These neural representations were evident from the first clicks, with all EBs and LB1 showing significant decoding in the EEG response before the second click onset, although LB1's response peaked afterward (Fig. 5*A*). Because our behavioral probes started at two clicks, the decoding significance onset does not tell us whether that information was perceptually available to the observers. However, decoding peaks in the first-click epoch showed a strong monotonic relationship with overall task accuracy for all four blind echolocators and the SC group (Spearman *ρ* = 1.0; *N* = 5; Fig. 5*C*), linking early neural discriminability with task performance. Together with the observed improvements across additional clicks, this pattern indicates that echolocation performance reflects both sequential integration and the quality of early sensory representations. Individuals with stronger initial neural differentiation exhibited superior behavioral performance, suggesting that early encoding quality may constrain subsequent decision accuracy, while later clicks refine or stabilize the emerging perceptual estimate. This pattern is consistent with the idea that echolocation proficiency reflects both rapid extraction of spatial cues and efficient use of sequential evidence, rather than reliance on either process alone.

Decoding of the last two clicks (Fig. 5*B*) showed persistent but attenuated decoding accuracy in EB participants relative to the first-click epoch. This temporal profile is compatible with a transition from early stimulus-driven sensory encoding toward later decision-related neural states as the sequence unfolds. Population-level recordings indicate that perceptual decisions can involve a shift between two dynamical regimes: an initial phase dominated by sensory input followed by a later phase governed by choice-related dynamics (Bondy et al., 2025; Luo et al., 2025). Within this framework, reduced discriminability at later clicks does not necessarily reflect weaker sensory representation but may instead indicate that neural activity has shifted from encoding stimulus laterality to representing the emerging decision itself.

### Sequence-position–dependent neural state dynamics

The ordinal-position decoding analysis (Fig. 6) quantified how neural population dynamics for successive clicks differed from those elicited by the first click, providing an index of how brain states changed across repeated stimulation. Because the first click marks the transition from baseline to stimulus-driven processing, the contrast between Click 1 and Click 2 would be expected to produce the largest change in neural state. Consistent with this prediction, Click 1 versus 2 decoding was highest across participants, near ceiling levels throughout the epoch in EB echolocators.

Across subsequent clicks, however, the relationship to Click 1 diverged between preclick and postclick intervals. Preclick decoding relative to Click 1 declined systematically as click order increased, becoming indistinguishable from the Click 1 baseline by later click-ordinal positions (Clicks 5–11, participant-dependent), even though stimulus-evoked responses remained robustly decodable. This dissociation suggests that while sensory responses to each click remained discriminable, the ongoing neural state between clicks became progressively more similar across later sequence positions.

Summing above-chance preclick decoding values across clicks yielded the trajectory shown in Figure 6*F*. These trajectories resembled the behavioral threshold profiles (Fig. 3): EB1 showed an early rise followed by stabilization, EB2 and EB3 exhibited more gradual increases before plateauing, and LB1 and SC showed little systematic change. This correspondence suggests that sequence-position–dependent neural state dynamics differences covary with behavioral dynamics across clicks. Importantly, because preclick signals are measured before stimulus onset, these effects do not necessarily reflect spatial encoding per se and may instead arise from stimulus-related state changes such as adaptation, expectation, or attentional states. Nevertheless, their systematic relationship with behavior suggests they index task-relevant neural processes engaged during sequential sampling. Consistent with this interpretation, model comparisons suggest that the brain progressively incorporates information across clicks.

### Computational model comparison constrains candidate readout policies

To test whether behavioral improvements reflected integration of sequential evidence rather than reliance on isolated samples, we compared trial-level computational models linking EEG-derived metrics to behavioral accuracy. We evaluated alternative readout rules corresponding to distinct candidate decision policies, spanning integrative and nonintegrative regimes. This approach allowed us to assess how sequential echo information may be used by the perceptual system without assuming a particular neural implementation. Within the echolocation literature, improvements with repeated clicks have been documented behaviorally (Thaler et al., 2018), but the computational principles underlying these gains have remained largely unresolved. Here, models implementing integrative readout rules provided the best account of behavior across cross-validated metrics, whereas individual participants were best described by distinct readout profiles. EB2 was better explained by an additive accumulation rule, whereas EB3 was better captured by a rate-sensitive readout reflecting sensitivity to variation of neural evidence. These findings indicate that improvements with additional clicks are not well explained by selection of a single optimal sample within the tested model space but instead are more consistent with the progressive combination of information across clicks. Even in EB1, whose performance was near ceiling, softmax sensitivity analyses did not support exclusive reliance on a single early click.

This heterogeneity indicates that expert echolocators do not rely on a single canonical decision rule but instead exhibit observer-specific readout profiles. Such variability may relate to differences in experience, age, training history, or the extent to which echolocation is used alongside other navigation tools. Together, these findings constrain plausible decision mechanisms, suggesting that echolocation decisions are more consistent with graded integration than with single-sample readout under the tested readout models.

### Potential of a sequence- and time-resolved approach to future work

The temporal dynamics observed in blind echolocators include a variety of signal trends: a strong initial response, attenuating decoding for each click, as well as signatures consistent with persistent and accumulating representations across clicks. These may reflect dissociable signatures of sensory- and decision-related processing, a nested cascade in which echoacoustic features are rapidly extracted, integrated, and refined into coherent spatial percepts. Future research using multimodal neuroimaging could combine spatial and temporal precision (Ritter and Villringer, 2006; Cichy and Oliva, 2020; Lowe et al., 2022) to help identify the specific cortical circuits involved and clarify how these networks evolve through training and experience. Our time-resolved approach may also be applied to studies of more complex echo scenes and objects, better approximating real-world scenarios, and eventually integrate more ecologically valid aspects such as head movements or full-body mobility in virtualized environments (Gramann et al., 2010; Miyakoshi et al., 2021). Finally, granular examination of sequential click processing dynamics would help identify behavioral and neural underpinnings of expertise, moving past presumptions based on visual experience and self-report. As sighted observers can readily perform (or learn) echolocation to various extents, characterizing echolocation expertise in terms of sensitivity to spatial acoustic cues, efficiency of integration across time/samples, scene-analytic processing, etc. can more rigorously inform training interventions (Norman et al., 2021; García-Lázaro and Teng, 2025) and serve as a promising avenue for revealing generalizable principles of perceptual learning and sensory compensation.

### Summary and conclusion

The present study provides, to our knowledge, the first behavioral and neural characterization of individual click dynamics and their integration over repeated samples. The enhanced localization performance and early neural encoding in EB echolocators are consistent with experience-dependent cortical adaptation to sensory deprivation. Our results may inform training programs aimed at enhancing echolocation in blind novices, as well as the development of assistive technologies that leverage temporal sampling strategies to support spatial perception. More fundamentally, this work highlights graded integration as a candidate computational principle in human echolocation, showcases the remarkable flexibility of the brain's perceptual systems in the absence of vision, and demonstrates a novel paradigm for future echolocation research.

## Synthesis

Reviewing Editor: David Schoppik, New York University Grossman School of Medicine Decisions are customarily a result of the Reviewing Editor and the peer reviewers coming together and discussing their recommendations until a consensus is reached. When revisions are invited, a fact-based synthesis statement explaining their decision and outlining what is needed to prepare a revision will be listed below. The following reviewer(s) agreed to reveal their identity: NONE.

Major point 1 - evidence accumulation vs alternative accounts

The manuscript interprets the observed improvements in performance with increasing numbers of clicks as reflecting evidence accumulation. However, no formal accumulation model is fitted to the data, nor are competing models (e.g., sequential sampling vs. single best sample) directly tested. The logic rests primarily on the fact that a standardised click-echo is repeated, and performance improves with more repetitions. While this pattern is certainly consistent with accumulation, it could also be explained by alternative accounts - for instance, that additional repetitions increase the probability of optimally encoding the stimulus on a given trial (e.g., across variations in lapses of attention or fluctuations in sensory encoding). Currently, we don't think that the manuscript shows convincingly that the results reflect evidence accumulation specifically.

Major point 2 - validity of the decoding analysis of clicks

The use of −200 to +550 ms click epochs is acceptable in principle, but it creates a bias or asymmetry when directly comparing click 1 with other clicks in the sequence: the "pre-click" window for click 1 is a true prestimulus baseline, whereas for later clicks it may contain lingering activity from the previous click or anticipatory dynamics. This is important because the authors interpret above-chance pre-click decoding as evidence of accumulated spatial representations, but this could also result from a number of confounding factors. It is noteworthy that the LB individual also shows very high decoding accuracy despite being behaviourally very poor at the task. What about the sighted controls, who weren't able to behaviourally do this task - do they also show the same decoding results? If so, it would suggest that the results shown in e.g. figure 6 are not meaningful but instead driven by an artefact of the experimental design and statistical analysis.

WeI are not sure of the rationale for grouping clicks by n and n-1 positions in their respective sequences for the EEG decoding analysis. We would not expect the evoked activity to be similar for clicks 11 and 5 for many reasons, so it doesn't make sense to me to group the clicks in this way. For comparing early vs late click decoding, is it not better to simply group by ordered position in the sequence? We also found it slightly unclear how this distinction was handled in 2-click trials, where the first two and last two clicks are identical. Please clarify how this was handled and consider whether this affects the results.

Major point 3 - poor decoding accuracy for later clicks compared to early clicks

This point is related to the previous one. The manuscript reports that EEG decoding accuracy for echo laterality is stronger for the first two clicks and attenuated for the last two clicks, which the authors attribute to repetition suppression. We are not fully convinced by this explanation, since repetition suppression typically refers to reductions in response magnitude, whereas the analysis here concerns the pattern of neural activity. Moreover, if anything, one might expect that later clicks would yield more discriminable left-right patterns, in line with the observed behavioural improvement with increasing numbers of clicks.

A more fundamental concern, however, is that the decoding analyses focus only on comparisons between click 1 and later clicks (e.g., click 2, 5, 8, 11). This design makes it difficult to know what the reduced decoding accuracy for later clicks actually reflects. The authors indirectly suggest that it demonstrates accumulation, but because the analysis is anchored only on click 1, we are left to infer whether the effect is indeed due to accumulation or some other factor. Taken together with our previous point on how the click epochs are defined, we am not convinced that the current results provide unambiguous evidence for accumulation.

Some addition comments:

- Some of the details in fig 1 a are difficult to see

## Author Response

Dear Dr. Schoppik, We thank you and the reviewers for the careful and insightful evaluation of our manuscript, "Neural and behavioral correlates of evidence accumulation in human click-based echolocation." We appreciate the time and thought invested in the review process and are grateful for the constructive comments, which have strengthened the rigor and clarity of the work. In response, we have substantially revised the manuscript. Specifically, we have expanded and clarified some of our existing analyses, added new formal comparisons of accumulative vs. alternative decision readout rules (SUM, MAX, SOFTMAX, SLOPE), revised the Abstract and Introduction to clarify the theoretical framework, and expanded the Discussion. Below, we provided detailed, point-by-point responses describing the corresponding revisions.

Major 1 - Evidence accumulation vs alternative accounts The manuscript interprets the observed improvements in performance with increasing numbers of clicks as reflecting evidence accumulation. However, no formal accumulation model is fitted to the data, nor are competing models (e.g., sequential sampling vs. single best sample) directly tested. The logic rests primarily on the fact that a standardised click-echo is repeated, and performance improves with more repetitions. While this pattern is certainly consistent with accumulation, it could also be explained by alternative accounts - for instance, that additional repetitions increase the probability of optimally encoding the stimulus on a given trial (e.g., across variations in lapses of attention or fluctuations in sensory encoding). Currently, we don't think that the manuscript shows convincingly that the results reflect evidence accumulation specifically.

Response We thank the reviewers for raising this important concern. We agree that performance improvements with increasing numbers of clicks, taken alone, are consistent with, but do not uniquely demonstrate, evidence accumulation. As noted, similar behavioral patterns could arise from repetition-based alternatives such as reliance on a single optimally encoded sample or trial-to-trial sensory fluctuations.

To address this directly, the revised manuscript now includes trial-level model comparisons that explicitly test competing decision readout rules (new Figure 7). We fit five logistic regression models predicting single-trial accuracy. The baseline model included only log-transformed click number, capturing the observed relationship between repetitions and performance. We then compared four augmented models, each adding one EEG-derived predictor constructed from click-wise responses using a different readout rule.

These predictors span three classes of candidate readout policies: integration-consistent (SUM and SLOPE), non-integrative (MAX), and intermediate or hybrid (SOFTMAX). SUM reflects cumulative integration across clicks, SLOPE captures systematic change across the sequence, MAX represents reliance on the strongest single click, and SOFTMAX implements graded weighting across clicks within a trial. While these models do not implement explicit generative accumulation dynamics, they directly test whether behavior is better explained by readout rules consistent with integration versus single-sample alternatives. To ensure that results were not driven by correlations between neural signal magnitude and repetition count, all EEG-derived predictors were residualized with respect to log-transformed click number before model fitting. This isolates trial-to-trial neural variability independent of sequence length. Models were evaluated using AICc and cross-validated predictive performance. Changes in AUC (ΔAUC) and prediction error (ΔRMSE) relative to baseline quantified the out-of-sample improvement. For each participant, the best-supported readout profile was determined based on converging metrics.

As shown in Figure 7, among early-blind echolocators with sufficient behavioral variability, model comparison favored integrative decision rules over non-integrative accounts. EB2 was best characterized by cumulative integration (SUM), whereas EB3 was best explained by SLOPE. EB1's near-ceiling performance (2 errors in 400 trials) limited inferential comparisons; results for this participant are therefore reported descriptively. In contrast, the late-blind participant and sighted control group showed no reliable improvement beyond the baseline behavioral model.

These analyses, detailed in the revised manuscript, provide a formal comparison between integrative and non-integrative accounts at the level of decision readout. Within the tested model space, behavior in expert echolocators was more consistent with graded integration across clicks than with reliance on a single optimal sample. Importantly, readout profiles differed across individuals, consistent with prior work showing variability in perceptual decision strategies across observers.

2 - Validity of the decoding analysis of clicks The use of −200 to +550 ms click epochs is acceptable in principle, but it creates a bias or asymmetry when directly comparing click 1 with other clicks in the sequence: the "pre-click" window for click 1 is a true prestimulus baseline, whereas for later clicks, it may contain lingering activity from the previous click or anticipatory dynamics. This is important because the authors interpret above-chance pre-click decoding as evidence of accumulated spatial representations, but this could also result from a number of confounding factors. It is noteworthy that the LB individual also shows very high decoding accuracy despite being behaviourally very poor at the task. What about the sighted controls, who weren't able to behaviourally do this task - do they also show the same decoding results? If so, it would suggest that the results shown in e.g., Figure 6 are not meaningful but instead driven by an artefact of the experimental design and statistical analysis.

We are not sure of the rationale for grouping clicks by n and n-1 positions in their respective sequences for the EEG decoding analysis. We would not expect the evoked activity to be similar for clicks 11 and 5 for many reasons, so it doesn't make sense to me to group the clicks in this way. For comparing early vs. late click decoding, is it not better to simply group by ordered position in the sequence? We also found it slightly unclear how this distinction was handled in 2-click trials, where the first two and last two clicks are identical. Please clarify how this was handled and consider whether this affects the results.

Response We thank the reviewers for raising these concerns regarding the EEG decoding analyses. We agree that careful interpretation is required when comparing neural responses across clicks within a sequence, particularly with respect to baseline definition, click grouping, and the relationship between decoding performance and behavior.

Baseline asymmetry and interpretation of pre-click decoding As the reviewer notes, the pre-click window for later clicks may contain residual or anticipatory activity that is not present in the true prestimulus baseline preceding Click 1. This asymmetry was inherent to the design and motivated the analysis. We focused on the pre-click window (−200 to 0 ms) to characterize how the ongoing neural state evolves across the sequence, rather than to isolate purely stimulus-evoked responses, making it a reasonable candidate window for examining persistent or sequence-dependent signal changes. The analysis does not explicitly differentiate residual or anticipatory contributors to the signal but tracks the progression of how "different" (i.e., decodable) the pre-click windows are from the original baseline. As the analysis in Fig. 6 D-E shows, for LB1 and SC, reliable baseline discriminability was limited to the earliest comparison (Click 1 vs. 2) and diminished for later clicks, suggestive of a brain state similar to that prior to any clicks. Additionally, the click-wise progression of that signal, shown in Fig. 6 F-G, follows a cumulative and plateauing pattern similar to the click-wise progression of behavioral accuracy shown in Figs. 2-3. In the revised manuscript, we do not interpret the pre-click decoding analyses in Fig. 6 as direct evidence of accumulation but frame them as describing sequence-dependent neural dynamics (which may include carry-over, expectation-related, or preparatory processes) that evolve similarly to behavioral performance. These analyses are now accompanied by trial-level modeling that formally compares candidate decision readout accounts linking EEG-derived click-wise evidence to behavioral performance (Fig. 7).

Decoding in late-blind and sighted control participants The late-blind participant (LB1) exhibited reliable decoding despite relatively poor behavioral performance in this task. Importantly, LB1 did not show systematic sequence-dependent baseline shifts comparable to early blind echolocators (Fig. 6), nor did EEG-derived models improve behavioral prediction beyond click count alone. We interpret this pattern as consistent with preserved sensory discriminability without evidence that click-wise information was effectively integrated into behavior.

We have also included new equivalent analyses in sighted controls, who performed at chance. Sighted participants showed reliable post-stimulus click-order decoding but weaker or absent pre-click effects relative to early blind echolocators (now reported; Fig. 6E-G), and EEG-derived predictors did not improve behavioral prediction beyond the baseline click-count model. Thus, while click-order decoding can occur in the absence of successful echolocation and is not sufficient on its own to establish accumulation, this analysis nevertheless reveals systematic group differences: expert echolocators exhibited larger sequence-dependent neural dynamics than controls, consistent with the behavioral and modeling results, supporting the interpretation that these effects reflect differences in behaviorally relevant neural processing rather than a trivial consequence of the experimental design.

Rationale for early-late comparisons and handling of two-click trials Grouping the first two versus the last two clicks (click N and click N-1) was intended to contrast early encoding phases with later pre-decision stages within each trial, rather than to equate specific ordinal positions across conditions. We acknowledge that the late phase in longer sequences differs from that of shorter sequences. In response to reviewer comments regarding whether late-sequence decoding might be influenced by overlap with earlier clicks, we repeated the analysis excluding two-click trials and restricting the dataset to 5-, 8-, and 11-click sequences (Figure 5B in the revised manuscript, which replaces the previous version). This analysis minimizes overlap between early- and late-click decoding windows and focuses on clicks most temporally distant from the first two in each trial and therefore most likely to capture sequence-dependent effects prior to the behavioral response. As shown in Figure 1, the overall pattern remained qualitatively similar: early blind participants continued to show temporally structured decoding during the final clicks, whereas sighted controls remained nonsignificant. Minor shifts in single-subject significance (e.g., EB1 is no longer significant; LB1 has reached significance) are likely attributable to differences in trial counts rather than substantive changes in neural dynamics.

Figure 1. Decoding accuracy time courses during the final two clicks of each sequence. Left panel: main analysis including all conditions (2-, 5-, 8-, and 11-click trials). Right panel: Control analysis excluding two-click trials and restricting the dataset to 5-, 8-, and 11-click sequences. Colored lines indicate individual blind participants; black line indicates sighted controls. Horizontal colored bars denote time windows of significant above-chance decoding (cluster-corrected, p < 0.05). For blind individuals, single-subject statistics were computed by comparing each participant's decoding curve to an empirical null distribution generated by applying SVM decoding to label-randomized trials, permuted 100 times.

Excluding two-click trials preserved the overall temporal structure and magnitude of late-phase decoding in early blind participants. Minor differences at the single-subject significance level reflect changes in trial counts rather than qualitative alterations in decoding dynamics 3 - Poor decoding accuracy for later clicks compared to early clicks This point is related to the previous one. The manuscript reports that EEG decoding accuracy for echo laterality is stronger for the first two clicks and attenuated for the last two clicks, which the authors attribute to repetition suppression.

We are not fully convinced by this explanation, since repetition suppression typically refers to reductions in response magnitude, whereas the analysis here concerns the pattern of neural activity. Moreover, if anything, one might expect that later clicks would yield more discriminable left-right patterns, in line with the observed behavioural improvement with increasing numbers of clicks. A more fundamental concern, however, is that the decoding analyses focus only on comparisons between click 1 and later clicks (e.g., click 2, 5, 8, 11). This design makes it difficult to know what the reduced decoding accuracy for later clicks actually reflects. The authors indirectly suggest that it demonstrates accumulation, but because the analysis is anchored only on click 1, we are left to infer whether the effect is indeed due to accumulation or some other factor. Taken together with our previous point on how the click epochs are defined, we am not convinced that the current results provide unambiguous evidence for accumulation.

Response We thank the reviewer for this important point. The original reference to "repetition suppression" was imprecise and has been removed. As the reviewers noted, repetition suppression typically refers to reductions in response magnitude, whereas multivariate decoding reflects changes in the pattern of neural activity. Consistent with our behavioral results, improved performance with increasing number of clicks reached a plateau, indicating that greater behavioral accuracy does not necessarily imply stronger instantaneous discriminability at later clicks. Once sufficient information has been sampled, neural activity may reflect stabilization of the emerging decision rather than continued increases in sensory separability (Bondy et al., 2025; Luo et al., 2025). Under this interpretation, the reduced late-click decoding observed in our data is compatible with a transition from early sensory toward decision-related processing stages.

At the same time, decoding comparisons anchored to Click 1 cannot distinguish accumulation from alternative explanations. We therefore present these analyses as descriptive demonstrations of sequence-dependent representational change, not as inferential evidence for accumulation. These considerations motivated the complementary trial-level modeling analysis, which directly compares competing candidate decision rules linking EEG-derived click-wise signals to behavioral performance (Fig. 7).

Minor:

1. Some of the details in Fig. 1a are difficult to see Response Thanks for highlighting this. We have increased the font size and the resolution of Figure 1.

We thank you for your time and consideration.

REFERENCES Bondy, A. G., Charlton, J. A., Luo, T. Z., Kopec, C. D., Stagnaro, W. M., Venditto, S. J. C., Lynch, L., Janarthanan, S., Oline, S. N., Harris, T. D., &Brody, C. D. (2025). Brain-wide coordination of internal signals during decision-making. In bioRxivorg. https://doi.org/10.1101/2024.08.21.609044 Luo, T. Z., Kim, T. D., Gupta, D., Bondy, A. G., Kopec, C. D., Elliott, V. A., DePasquale, B., &Brody, C. D. (2025). Transitions in dynamical regime and neural mode during perceptual decisions. Nature, 646(8087), 1156-1166.
